# Left atrial appendage morphology and hemodynamics: potential new markers for individualized thromboembolic risk assessment in atrial fibrillation

**DOI:** 10.3389/fcvm.2026.1924120

**Published:** 2026-10-06

**Authors:** Bingxin Liu, Xianyong Liu, Changlin Zhang, Henan Zhao

**Affiliations:** 1 Department of Joint and Sports Medicine, The Second Hospital of Dalian Medical University, Dalian, Liaoning, China; 2 Department of Cardiology, Xinhua Hospital Affiliated to Dalian University, Dalian, Liaoning, China; 3 Department of Pathophysiology, College of Basic Medical Sciences, Dalian Medical University, Dalian, Liaoning, China

**Keywords:** atrial fibrillation, digital twin, hemodynamics, left atrial appendage, thromboembolism

## Abstract

**Background:**

Traditional CHA₂DS₂-VASc scoring has well-recognized limitations in thromboembolic risk assessment for atrial fibrillation (AF), particularly in low-score patients who nevertheless thrombose, high-score event-free individuals, cancer patients, and post-ablation cohorts. The left atrial appendage (LAA) is the source of 98% of thrombi in non-valvular AF and exhibits marked inter-individual morphological and hemodynamic variability that traditional scores completely ignore.

**Aims:**

This narrative review critically evaluates current evidence on LAA morphology, hemodynamics, computational fluid dynamics/fluid-structure interaction (CFD/FSI) studies, and digital twin technology, and proposes a mechanism-driven, individualized risk assessment framework for AF-related thromboembolism.

**Methods:**

A literature search was conducted in PubMed/MEDLINE and Web of Science (1990–May 2026) using combinations of keywords related to LAA morphology, hemodynamics, AF, and thromboembolism. Priority was given to landmark original studies, high-quality observational studies with multivariable adjustment, meta-analyses, and contemporary CFD/digital twin studies. A narrative synthesis was adopted owing to heterogeneity in study designs and outcomes.

**Results:**

LAA morphological subtypes are associated with long-term stroke risk, with chicken-wing being protective (OR 0.21, 95% CI 0.05–0.91) and cauliflower carrying the highest risk (OR 8.02, 95% CI 0.92–27.86). However, risk is highly context-dependent, with chicken-wing unexpectedly increasing peri-ablation thromboembolic risk (OR 7.2, 95% CI 1.35–38.33) and windsock morphology independently predicting post-ablation AF recurrence (OR 2.72, 95% CI 1.21–6.12). Importantly, traditional morphological classification does not correlate directly with blood stasis (*p* = 0.365), whereas quantitative geometric parameters such as trabecular number, normalized stroke volume, and ostial area are far more sensitive predictors (R² = 74%). Digital twins integrating multimodal clinical, morphological, and hemodynamic data identify a high-risk phenogroup with a 70% thrombus history rate.

**Conclusion:**

Based on these findings, we propose a practical two-tier assessment framework combining basic morphological classification with multimodal refined evaluation (digital twin) for intermediate-/high-risk or clinically challenging cases. This framework represents a paradigm shift from static clinical scores toward mechanism-driven, individualized risk management. Prospective validation of this approach is now warranted.

## 1 Introduction: multiple clinical conundrums of traditional scores and the potential role of the LAA

Atrial fibrillation (AF) is the most common cardiac arrhythmia, accounting for approximately a quarter of ischemic strokes (1–3). Oral anticoagulation (OAC) is the cornerstone for preventing AF-related stroke (4, 5). The CHA₂DS₂-VASc score (6–8) is widely used but has well-recognized limitations in low-score patients who thrombose, high-score event-free individuals, cancer patients, and post-ablation cohorts (9–16). The 2024 ESC guidelines updated the score to CHA₂DS₂-VA (4, 17), yet these scores remain static models based solely on demographic and clinical risk factors.

Di Biase et al. found that among low-risk CHADS₂ 0-1 patients, non-chicken-wing left atrial appendage (LAA) morphology was associated with a 10-fold higher prior stroke/TIA prevalence (4.6% vs. 0.7%, adjusted OR 10.1, 95% CI 1.25–79.7) (9). The same study showed that prior stroke/TIA was only 4% in chicken-wing but higher in other morphologies (cactus 12%, windsock 10%, cauliflower 18%), suggesting that some high-score patients may remain event-free due to a protective LAA (9).

In cancer patients, drug interactions, thrombocytopenia, and hypercoagulability impair traditional scores (10–13). Direct assessment of the LAA provides valuable risk information specifically in cancer patients. Transesophageal echocardiography (TEE)-detected LAA abnormalities strongly predict thromboembolic events in this population (18). LAA thrombus formation has been documented in cancer patients despite adequate anticoagulation, underscoring the importance of dedicated LAA evaluation in this high-risk population (12, 19).

After ablation, AF burden is reduced but cardiovascular risk persists (age, hypertension, etc.). Traditional scores do not account for anatomical and functional remodeling of the LAA. Reduced LAA emptying velocity predicts AF recurrence, which in turn increases thromboembolic risk (14). A meta-analysis confirmed that reduced LAA emptying velocity, increased LAA volume, and reduced LAA ejection fraction are all significantly associated with post-ablation recurrence (15). Specifically, pre-ablation LAA emptying velocity ≤52.3 cm/s increases relapse risk by nearly 50% (20), larger LAA volume independently predicts recurrence (21), left atrial spontaneous echo contrast (SEC) confers a 3.5-fold higher thromboembolic risk (22), incidental LAA electrical isolation may increase stroke risk unless anticoagulation or LAA occlusion is continued (23), and even “protective” chicken-wing morphology paradoxically elevates peri-procedural risk (adjusted OR = 7.2) (24). These findings underscore the need to integrate LAA specific parameters into post ablation risk stratification, moving beyond static clinical scores.

A common reason for these conundrums is that traditional scores ignore the LAA, the source of up to 98% of atrial thrombi (9, 22, 25). LAA size, shape, and hemodynamics vary greatly among individuals, yet current traditional risk scoring systems have not incorporated these morphological features (19). Multiple studies have confirmed an association between LAA morphology and long-term stroke risk (9, 26). However, the relationship is context-dependent. Kosiuk et al. reported that chicken-wing LAA, protective long-term, becomes associated with higher peri-ablation risk (adjusted OR 7.2, 95% CI 1.35–38.33, *p* = 0.021) (24). Lin et al. found windsock morphology independently predicts 1-year post-ablation AF recurrence (adjusted OR 2.72, 95% CI 1.21–6.12, *p* = 0.016) (27).

Beyond morphology, hemodynamics are critical for thrombogenesis. Saiz-Vivó et al. developed a digital twin integrating clinical, morphological, and hemodynamic data, identifying a high-risk phenogroup with 70% thrombus history (19). Musotto et al. found no direct correlation between morphological class and blood stasis (*p* = 0.365), suggesting quantitative geometric parameters (trabecular number, normalized stroke volume, ostial area) are more sensitive (28). Masci et al. showed that LAA geometric characteristics (length, tortuosity, ostial area, volume) significantly influence stasis, and even qualitatively simple LAA can carry high risk (29). These findings explain clinical inconsistencies among studies and provide a rationale for selecting input features for digital twin models.

This narrative review integrates the above evidence to argue for a paradigm shift from static clinical risk scores toward mechanism-driven, individualized risk assessment that incorporates patient-specific LAA morphology, hemodynamics, and treatment response heterogeneity. It explores whether LAA morphology and hemodynamics could serve as potential new markers for individualized thromboembolic risk assessment in AF, and offers evidence-based management strategies for certain special populations described above.

## 2 Methods

### 2.1 Review design and scope

This is a narrative review. Its quality and transparency follow the SANRA criteria (30) regarding literature search, referencing, scientific reasoning, and data presentation.

### 2.2 Literature search

PubMed/MEDLINE and Web of Science (1990–May 2026) were searched using combinations of (“left atrial appendage” OR “LAA”) AND (“morphology” OR “hemodynamics” OR “stasis”) AND (“atrial fibrillation”) AND (“thromboembolism” OR “stroke” OR “thrombosis”). Additional terms included (“cancer” OR “malignancy” OR “tumor”), (“ablation” OR “post-ablation” OR “after ablation”), and (“CHA2DS2-VASc” OR “CHA2DS2-VA”). Only English-language articles were considered. Two authors (X. Liu and C. Zhang) independently screened titles/abstracts; discrepancies were resolved by a third author (H. Zhao). Duplicates were removed, and reference lists were manually screened.

### 2.3 Study selection and synthesis

Priority was given to landmark original studies, high-quality observational studies with multivariable adjustment, meta-analyses, and contemporary computational fluid dynamics (CFD)/digital twin studies. Owing to heterogeneity in designs and outcomes, a narrative synthesis was adopted, organizing evidence thematically around LAA morphology, hemodynamics, and multimodal risk stratification.

### 2.4 Referencing and reporting of effect estimates

Effect estimates are reported with 95% confidence intervals where available. Tables follow the same principle.

## 3 Left atrial appendage: a potential key structure for thrombus formation and risk assessment

### 3.1 LAA anatomy and thrombogenic mechanisms

The left atrial appendage is an embryological remnant on the anterolateral left atrial wall, adjacent to the left superior pulmonary vein, left circumflex artery, and left phrenic nerve (9). During sinus rhythm, the LAA contributes to left atrial reservoir function, volume and pressure modulation, partly through a dense network of pectinate muscles that generate contractile forces aiding its filling and emptying (19).

In atrial fibrillation, the organized contraction of the LAA is lost, leading to reduced contractility and incomplete blood emptying (29). Consequently, left atrial appendage emptying velocity (LAAev) is significantly reduced, creating flow stagnation, vortex formation, and disrupted washout (31, 32). As early as 1999, Al-Saady et al. described that in AF, LAA flow may become absent or severely reduced, with some patients showing no identifiable flow waves (33).

AF also triggers a hypercoagulable state involving circulating coagulation factors, platelet activation, inflammatory markers, oxidative stress, and endothelial dysfunction (10). All three components of Virchow's triad (abnormal blood stasis, endothelial damage, hypercoagulability) are satisfied in AF (34). Coagulation system activation is extensive within the LAA, where markers such as P-selectin and von Willebrand factor (vWF) antigen are significantly elevated compared with systemic circulation, suggesting a local prothrombotic microenvironment (35). The HEART-CLOT study further demonstrated a local prothrombotic state in the LAA of AF patients, characterized by the formation of more compact fibrin clots and prolonged clot lysis time compared with peripheral blood (36). These pathophysiological alterations transform the LAA into the predominant thrombogenic site, harboring >90% of left atrial thrombi in non-valvular AF (22, 25).

### 3.2 Morphological classification and quantitative evidence for long-term stroke risk

Di Biase et al. (9) analyzed LAA morphology by CT/MRI in 932 AF patients and classified the LAA into four distinct configurations with different thrombotic risks: Chicken Wing, Cactus, Windsock, and Cauliflower. Among these, the chicken-wing type is the most prevalent (48%), characterized by an obvious bend or fold in the proximal or middle part of the dominant lobe. Multivariate regression analysis correcting for CHADS₂ score, gender, left atrial size, and AF types showed that the chicken-wing morphology independently reduced the risk of stroke/TIA by 79% (OR 0.21, 95% CI 0.05–0.91, *p* = 0.036) (9). The other morphologies carried significantly higher risks (Table 1), suggesting that LAA morphology itself is an independent predictor of stroke beyond traditional clinical scores.

**Table 1:** LAA morphology types and thrombotic risk [data from Di Biase et al. (9)].

LAA morphology	Proportion	prior stroke/TIA	OR vs. chicken-wing (95% CI)	*p* value
Chicken-wing	48% (451/932)	4%	Reference	—
Cactus	30% (278/932)	12%	4.08 (1.04–17.27)	0.046
Windsock	19% (179/932)	10%	4.50 (1.89–22.50)	0.038
Cauliflower	3% (24/932)	18%	8.02 (0.92–27.86)	0.056

A meta-analysis by Lupercio et al. (26) (8 studies, 2,596 patients) further confirmed that the chicken-wing morphology was associated with a 54% lower thromboembolic risk compared to non-chicken-wing morphologies (OR 0.46, 95% CI 0.36–0.58).

From a clinical imaging perspective, both CT and MRI are established modalities for LAA morphological characterization, though their roles differ from TEE. According to the 2024 ESC guidelines for AF management, TEE remains the primary imaging modality for excluding LAA thrombus before cardioversion (4). For patients undergoing ablation, imaging to exclude thrombus should be considered for those at high thromboembolic risk. CT is valuable for pre-procedural LAA morphology assessment prior to AF ablation or LAA occlusion, given its high spatial resolution (sub-millimeter), rapid acquisition, and three-dimensional multiplanar reconstruction capabilities, which are essential for defining LAA orifice size, depth, and morphology (4, 9). MRI offers complementary value for LAA assessment with the advantages of no ionizing radiation and superior soft-tissue contrast; it is particularly useful when radiation exposure is a concern, such as in young patients or those requiring repeated imaging, or when concomitant evaluation of atrial fibrosis is needed (4). The 2023 ACC/AHA/ACCP/HRS guidelines also acknowledge the utility of CT and MRI for LAA evaluation, particularly when TEE is inconclusive or contraindicated, highlighting their value in anatomical definition (37). Thus, while TEE remains the clinical standard for thrombus detection, CT and MRI are increasingly recognized as complementary tools with distinct strengths, including CT for anatomical definition and procedural planning, and MRI for tissue characterization and fibrosis assessment. These imaging modalities provide the morphological data that underpin the computational and digital twin approaches discussed in subsequent sections.

### 3.3 Differential impact across clinical scenarios

The relationship between LAA morphology and thromboembolic risk is not fixed but highly dependent on the clinical context. For example, during the peri-ablation period, Kosiuk et al. (24) performed a 1:4 matched case-control study in 2,570 AF patients undergoing catheter ablation, including 17 patients who developed thromboembolic events within 30 days. The results showed that the chicken-wing morphology accounted for 65% of the event group versus only 21% in controls (*p* < 0.001), with an adjusted OR of 7.2 (95% CI 1.35–38.33). The proposed mechanism is that the curved, folded structure of the chicken-wing LAA may protect against spontaneous large-thrombus release during long-term follow-up, but during ablation, microthrombi generated by catheter irrigation are easily trapped in its tortuous recesses and subsequently embolize. The authors also noted that other mechanisms, such as coagulation, endothelial function, or platelet activation, might be involved, but no sufficient data were available for validation, warranting further studies (24).

On the other hand, LAA morphology also influences long-term outcomes after ablation. Lin et al. (27) followed 463 first-time ablation patients for one year and found that the windsock morphology had the highest recurrence rate (48.3%), significantly higher than the chicken-wing (25.2%), cauliflower (20.8%), and cactus (18.2%) types (*p* = 0.017). Multivariable analysis confirmed that the windsock morphology was an independent predictor of AF recurrence (adjusted OR 2.72, 95% CI 1.21–6.12), indicating that these patients require more intensive rhythm monitoring and antiarrhythmic therapy after ablation.

### 3.4 Hemodynamic parameters and the interplay with morphological-structural factors

Local hemodynamic conditions inside the LAA are critical determinants of the likelihood of thrombus formation. The peak LAA emptying velocity measured by TEE is a classic marker of blood stasis. A velocity below 20 cm/s is associated with a significantly increased thrombotic risk, whereas a velocity of at least 40 cm/s is generally considered a low-risk threshold (31, 32, 38). Furthermore, Qureshi et al. (31) used MRI-based CFD to quantify the wall shear stress (WSS), oscillatory shear index (OSI), and endothelial cell activation potential (ECAP) on the LAA endocardial surface. Their study demonstrated that during AF, decreased WSS and increased OSI lead to elevated ECAP, which promotes endothelial dysfunction and thrombus formation. After successful catheter ablation, LAA flow velocity can increase by 24%–166%, while the area under the fibrin generation curve decreases by 51%–97% (31).

Beyond these established markers, previous fluid-structure interaction (FSI) studies have revealed that the loss of active LAA contractility is the dominant factor driving thrombogenic hemodynamics. Vella et al. (39) demonstrated that AF-induced impairment in contractility alone reduces shear strain rate (SSR) by approximately 50%, while chamber dilatation contributes an additional 30% reduction. Critically, rigid-wall models, a common simplification in earlier CFD studies, produced SSR values one order of magnitude lower than contractile models, indicating that neglecting wall motion substantially underestimates thrombotic risk (39). Under AF conditions, all morphological types exhibit similarly reduced SSR values and predicted risk levels, despite marked differences in their gross shape (40).

Importantly, hemodynamic parameters must be distinguished from morphological-structural factors, which are static anatomical features including LAA geometry (length, tortuosity, ostial area, volume), trabecular number and distribution, lobe architecture, and the presence of sudden bends or knees (40, 41). While global morphological classification (chicken-wing vs. non-chicken-wing) has been epidemiologically associated with stroke risk, FSI studies demonstrate that traditional morphological class does not correlate directly with blood stasis (*p* = 0.365) (40). Instead, local topological features, such as trabecular density, lobe depth, and bend angles, are more directly associated with regions of low SSR and thrombus formation (40). Computational studies of LAA inversion further reveal that trabecular structures act as “tie beams” connecting opposite walls, resisting deformation and influencing mechanical stability under abnormal loading conditions (41).

Recent advancements have moved beyond correlative risk assessment to simulate the actual process of thrombus formation, growth, and embolization within the LAA (42). Using a smoothed particle hydrodynamics (SPH) approach that tracks biochemical species (prothrombin, thrombin, fibrinogen, fibrin) and three platelet types (resting, activated, bound), this model demonstrated that thrombus formation initiates at the LAA tip, confirming clinical observations that the tip is the most thrombogenic region due to blood stagnation, and progressively expands through lobes and trabeculae (42). Critically, while the LAA tip is the site of origin, the risk of embolization is highest in regions with high recirculation near the orifice, where formed clots are more likely to detach and migrate (42). This distinction, between the site of thrombus formation (often structural/trabecular) and the site of embolization risk (often flow-driven), underscores the necessity of integrating both morphological and hemodynamic assessments in risk stratification.

The clinical implications of this distinction are clear. Fluid-dynamic parameters are modifiable; restoration of sinus rhythm by ablation increases LAA emptying velocity by 24%–166% (39). In contrast, morphological-structural parameters are largely fixed, except for progressive remodeling in chronic AF. Thus, fluid-dynamic assessment informs treatment response, while morphological assessment informs baseline risk. Current clinical risk scores (CHA₂DS₂-VASc and CHA₂DS₂-VA) incorporate neither category, representing a fundamental gap that the proposed digital twin-based framework aims to address.

### 3.5 Limitations of traditional morphological classification and the value of quantitative geometric parameters

Although the conventional four-type morphological classification is epidemiologically associated with stroke risk, recent CFD and FSI studies have revealed its inherent limitations. Musotto et al. (28) performed FSI analysis on 12 patient-specific LAA models derived from non-valvular AF patients (three from each morphological class) and found no statistical correlation between the traditional morphological classes and the quantitative blood stasis metric, defined as BSF5 (the LAA surface area exposed to a shear strain rate below 5 s⁻¹ throughout the entire cardiac cycle, *p* = 0.365). In contrast, the number of trabeculae (N_trabeculae) and the normalized stroke volume V* (defined as stroke volume divided by minimum volume during the cardiac cycle) were more sensitive predictors of thrombotic risk. The ratio N_trab/V* achieved an R² of 74%, significantly outperforming the LAA ostial velocity (R²=43%). This finding indicates that the fine internal structures of the LAA (e.g., trabeculae) and its global pumping function better reflect thrombotic risk than gross morphology. Moreover, Masci et al. (29) used CFD simulations with strictly controlled left atrial background geometry, varying only the LAA shape. They demonstrated that even a qualitatively simple, linear LAA can cause severe blood stasis and particle retention if it has a small ostial area, excessive length, or distal narrowing, and its thrombotic risk is no lower than that of a complex cauliflower-type LAA. Thus, quantitative geometric parameters (length, tortuosity, ostial area, volume, etc.) are the key determinants of stasis, and relying solely on a qualitative “simple/complex” classification is misleading.

Beyond their role in overcoming the limitations of traditional classification, these static geometric parameters carry independent predictive value for thrombotic risk, irrespective of hemodynamic conditions. They directly influence blood stasis as shown by CFD simulations (29), and they have been successfully incorporated into digital twin models for individualized risk stratification. For example, in the digital twin study by Saiz-Vivó et al. (19), LAA centerline length, ostial area, and LA volume were among the top features distinguishing high-risk from low-risk phenogroups. Therefore, while traditional morphological classification serves as a useful initial screening tool, accurate and individualized thrombotic risk assessment requires the integration of quantitative geometric parameters along with hemodynamic data. These parameters can provide a crucial basis for feature selection in digital twin models (19).

## 4 Digital twin and multimodal integration

### 4.1 Why do we need a digital twin?

The preceding sections have shown that LAA morphology and hemodynamics are closely associated with thrombotic risk. However, traditional morphological classification alone is insufficient for accurate risk stratification, and a tool that integrates multimodal data is lacking (11). Digital twin technology offers a possible solution: by fusing clinical, imaging, and hemodynamic data, it simulates patient-specific thrombogenic risk (11). Specifically, a digital twin can integrate quantitative geometric parameters (ostial area, left atrial volume, LAA centerline length, etc.) and hemodynamic indices (LAA particle count) with clinical features (BNP, MPV/PC, LVEF, renal function) within a unified framework – a direct translational application of the CFD/FSI findings.

### 4.2 Digital twin framework and key findings

Saiz-Vivó et al. (19) built a left atrial digital twin integrating multimodal data in 130 high-risk AF patients (mean CHA₂DS₂-VASc 4.0). They extracted LAA morphological parameters from CT, performed CFD simulations to obtain hemodynamic indices (e.g., number of particles remaining in the LAA), and combined clinical and laboratory data (BNP, MPV/PC, LVEF, etc.), totaling 47 features (19). Using unsupervised multiple kernel learning (MKL) and K-means++ clustering, they identified three phenogroups (0, 1, 2) (19).

The key results showed that the high-risk phenogroup (Phenogroup 2) had a 70% (21/30) prevalence of previous thrombosis, which was significantly higher than that in Phenogroup 1 (40%) and Phenogroup 0 (33%) (*p* = 0.006) (19). Table 2 presents the key characteristics of the three phenogroups.

**Table 2:** Key characteristics of the three phenogroups [the data comes from the research by saiz-vivó et al. (19)].

Feature	Domain	Phenogroup 1 (lower risk)	Phenogroup 2 (high risk)	*p* value
BNP (pg/mL)	Clinical	92.2 (59.5–129.1)	250.8 (211.8–318.9)	<0.01
LAA particle count	Hemodynamics	114.5 (70.3–177.3)	214 (193–247)	<0.01
LAA ostial area (mm²)	Morphology	349.8 (290.4–465.4)	701.6 (598.8–784.3)	<0.01
LA volume (mL)	Morphology	126.3 (101.8–139.8)	211.6 (164.4–247.9)	<0.01
LAA centerline length (mm)	Morphology	41.4 (36.1–45.4)	52.7 (44.0–55.6)	<0.01

SHAP analysis revealed that BNP level was the most important feature for distinguishing phenogroups, followed by ostial area, LA volume, centerline length, and tortuosity (19). In the supervised model directly predicting thrombus history, LAA particle count ranked first (19).

### 4.3 Potential clinical value of digital twins

Based on the above findings, digital twins may assist clinical decision-making in several aspects. For patients with low traditional scores but high-risk LAA morphology (e.g., cauliflower type, large ostial area), digital twins can help identify these “low-score but high-risk” individuals and assign them to the high-risk phenogroup. For patients with high traditional scores but protective LAA features (chicken-wing type with high flow velocity), digital twins can help confirm whether they are truly low risk, thereby supporting de-escalation of anticoagulation. In cancer patients, digital twins can integrate cancer-related variables (e.g., platelet count, renal function) with LAA parameters to more accurately assess the net clinical benefit of anticoagulation. For post-ablation patients, digital twins can dynamically evaluate the impact of reduced AF burden on LAA hemodynamics, providing individualized guidance for discontinuing oral anticoagulation.

### 4.4 A two-tier framework

We propose a conceptual two-tier framework for AF thromboembolic risk assessment. This framework is designed to translate the evidence synthesized in this review into a clinically actionable pathway, addressing the fundamental limitation that current risk scores completely ignore LAA-specific parameters.

The first tier is basic morphological classification, applicable to all AF patients. By CT or MRI, the LAA is rapidly classified into chicken wing, windsock, cactus, or cauliflower types, using known risk differences [cauliflower type carries the highest risk (9), while chicken wing type is protective 9] for initial screening. While morphological classification itself is not novel, its systematic integration into a routine clinical workflow represents a practical advance over current practice, where LAA morphology is rarely considered in risk stratification despite robust evidence of its association with stroke risk (9, 28).

Of note, the high-risk phenogroups identified by digital twin modeling share clinical characteristics (elderly patients with heart failure, COPD, and fall history) with patients whom a causal machine learning analysis of 744,190 real-world AF patients identified as more likely to benefit from LAA occlusion than from continued anticoagulation (43). This convergence between mechanism-driven digital twin phenogrouping and data-driven causal machine learning provides external validation that these high-risk profiles are clinically meaningful and may guide patient selection for LAAO. The alignment of two independent methodological approaches, one physics-based (digital twin) and one data-based (causal ML), strengthens the credibility of the identified high-risk phenogroups and supports their potential utility in clinical decision-making.

The second tier is multimodal refined evaluation (digital twin), intended for patients classified as intermediate or high risk in the first tier or for those in whom clinical decision making is difficult. This tier incorporates clinical features (BNP, MPV/PC, LVEF, renal function), quantitative morphological parameters (ostial area, LA volume, LAA centerline length), and hemodynamic parameters (LAA particle count, particle residence time), and outputs an individualized thrombotic risk probability and anticoagulation recommendation (Figure 1) (19). This tier represents the truly innovative component of our framework: it synthesizes clinical, morphological, and hemodynamic data into a patient-specific risk probability within a unified computational framework. This approach is not yet implemented in routine clinical practice and represents a paradigm shift from static clinical scores toward dynamic, mechanism-driven, individualized risk assessment.

**Figure 1:**
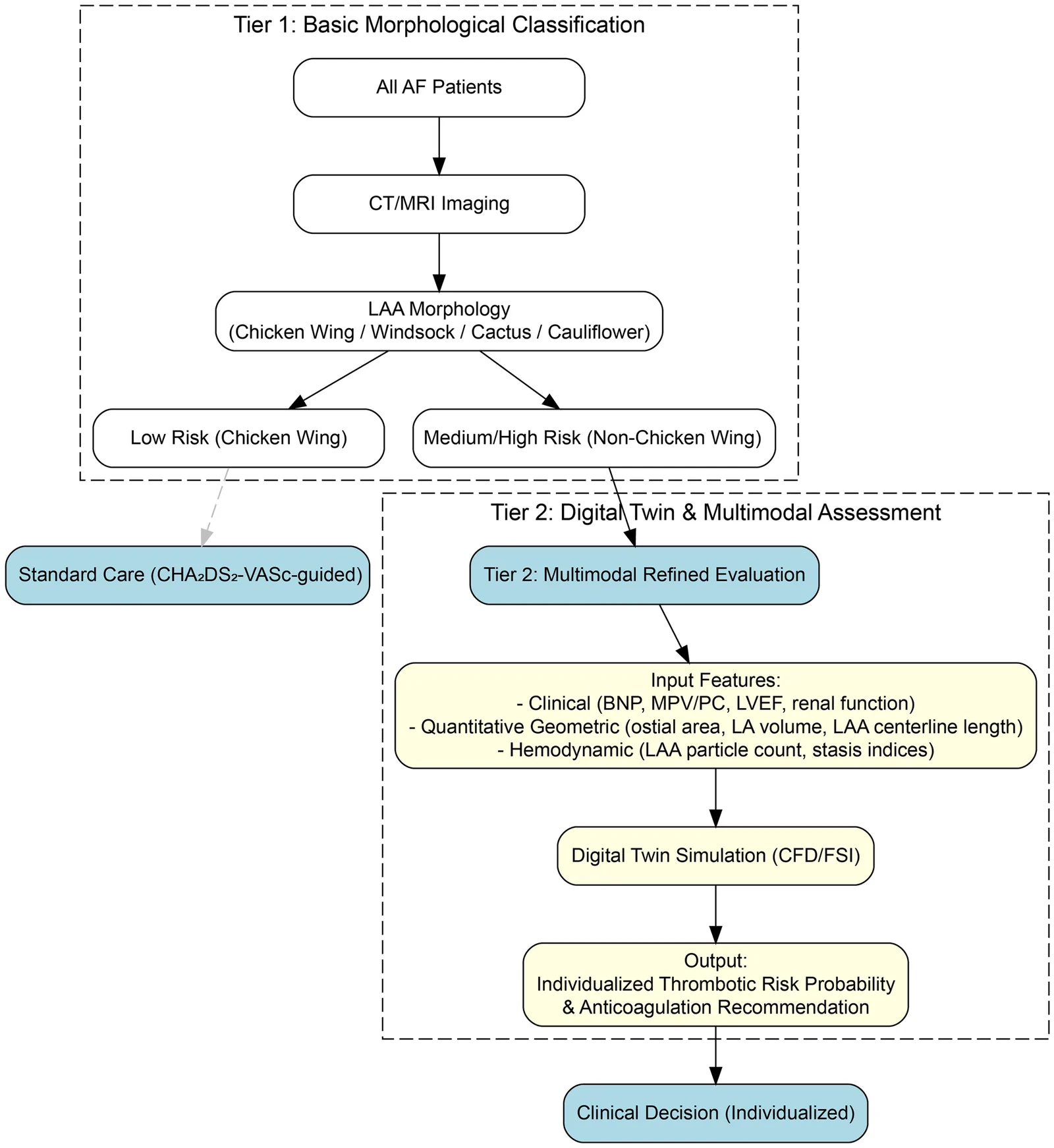
Schematic diagram of the two-tier assessment framework for thromboembolic risk in atrial fibrillation. Tier 1: basic morphological classification (CT/MRI) for initial screening in all AF patients. Low risk (chicken wing) can be managed with standard CHA₂DS₂-VASc-guided care (dashed arrow), while medium/high risk (non-chicken wing) proceed to Tier 2. Tier 2: multimodal refined evaluation using a digital twin, integrating clinical, quantitative geometric, and hemodynamic data to output individualized thrombotic risk probability and guide anticoagulation or LAA occlusion.

The framework should be viewed as hypothesis-generating rather than a ready-to-use clinical algorithm. It is intended to guide future prospective studies and clinical trial design by providing a structured approach for integrating multimodal LAA data into risk stratification. The innovation lies not in any single component, but in the integration of multiple data types, including morphology, hemodynamics, digital twin modeling, and machine learning, into a coherent, clinically actionable pathway that directly addresses the fundamental gap in current risk scores. Prospective validation of this framework is now warranted before its implementation in clinical practice.

## 5 Management strategies for special populations

### 5.1 The paradox of CHA₂DS₂-VASc score and thromboembolic risk

In clinical practice, the CHA₂DS₂-VASc score exhibits significant limitations. It fails to predict thromboembolic events in some low-risk patients (score 0–1), and it cannot explain the event-free status of certain high-risk individuals (score ≥4). These observations suggest that factors beyond traditional clinical variables, particularly LAA characteristics, may play a critical role in thromboembolic risk stratification (9, 44).

#### 5.1.1 Low-score patients with thromboembolic events

Even among patients with a low CHA₂DS₂-VASc score (0–1), traditionally considered at low risk for thromboembolism, residual stroke risk exists and varies substantially among individuals (16). In a Korean cohort of 542 non-valvular AF patients with a CHA₂DS₂-VASc score of 0 (mean age 49.9 years), the annual rate of stroke or systemic embolism was 0.78%/year (45). Independent predictors included age ≥50 years (HR 6.71, 95% CI 1.81–24.86), LVEDD ≥46 mm (HR 4.51, 95% CI 1.04–19.63), and non-paroxysmal AF (HR 5.58, 95% CI 1.62–19.18); patients with all three factors had an event rate of 4.21%/year (45).

Given these limitations, identifying additional risk markers beyond clinical variables is clinically relevant. Di Biase et al. (9) demonstrated that in the low-risk subgroup (CHADS₂ 0–1), non-chicken-wing LAA morphology was associated with a 10-fold higher prevalence of prior stroke/TIA (OR 10.1, 95% CI 1.25–79.7). However, reliance solely on morphological classification may underestimate risk. CFD/FSI studies showed no direct correlation between morphological classes and blood stasis (*p* = 0.365) (28); instead, quantitative geometric parameters (enlarged ostial area, excessive length) and hemodynamic parameters (reduced LAA emptying velocity, reduced emptying fraction) are more sensitive (19, 28, 29).

TEE studies established that reduced LAA emptying velocity increases thromboembolic risk. Fatkin et al. reported that a peak LAA velocity <35 cm/s was associated with a 28-fold increased odds of SEC (46). A velocity <20 cm/s is regarded as severely decreased and is often accompanied by dense SEC or LAA thrombus (47). In patients with LAA thrombus, the mean LAA emptying velocity was significantly lower than in those without thrombus (35 cm/s vs. 42 cm/s, *p* = 0.004) (47). LAA emptying fraction is another parameter reflecting LAA contractile function. Hwang et al. reported that patients with rheumatic mitral valve disease and atrial fibrillation had a markedly reduced LAA ejection fraction compared with non-rheumatic controls (13.5 ± 10.4% vs. 25.9 ± 12.9%), and those with LAA thrombus exhibited even lower values (48).

As for quantitative geometric parameters, although standardized clinical cutoffs are not yet available, CFD/FSI cohort data indicate that high-risk phenotypic groups typically exhibit a substantially enlarged LAA ostial area (e.g., >500 mm²), increased LAA length (e.g., >50 mm), or enlarged LA volume (e.g., >150 mL) (19). These observational reference values may inform multimodal assessment.

#### 5.1.2 High-score patients without thromboembolic events

The second paradox concerns patients with high CHA₂DS₂-VASc scores (e.g., ≥4) who remain free from thromboembolic events. Direct evidence for this phenomenon is limited, but the most plausible explanation is the presence of protective LAA features. Di Biase et al. demonstrated that chicken-wing LAA morphology independently reduces long-term stroke/TIA risk by 79% (OR 0.21, 95% CI 0.05–0.91) (9). Preserved LAA emptying velocity (≥40 cm/s) indicates low stasis (32, 38), and a small ostial area (<300 mm²) may further reduce thrombotic risk. These protective characteristics may offset the calculated high risk in some patients.

However, the absence of thromboembolic events does not imply zero risk, particularly in elderly patients. Even with protective LAA features, embolic sources may be extra-appendageal, especially aortic arch plaques (49–51).

#### 5.1.3 Multimodal assessment and clinical implications

Based on the above evidence, a multimodal assessment integrating LAA morphology, hemodynamics, and quantitative geometric parameters is recommended for patients with paradoxical risk profiles. For low-score patients with high clinical suspicion, TEE, CT, and CFD/FSI-derived parameters are necessary to identify otherwise overlooked high thrombotic risk. For high-score patients with protective LAA features, de-escalation of anticoagulation may be considered only after excluding extra-appendageal embolic sources. Individualized clinical judgment remains paramount, as dedicated randomized controlled trials (RCTs) are lacking.

### 5.2 Cancer patients

Cancer patients face a triple challenge of drug interactions between direct oral anticoagulants (DOACs) and CYP3A4/P-gp inhibitors, thrombocytopenia, and hypercoagulability (10). Traditional risk scores such as CHA₂DS₂-VASc have limited discriminative capacity in this population (10). Recent studies showed that the CHA₂DS₂-VASc score underestimates thromboembolic risk in cancer patients (13) and that its utility in cancer patients remains uncertain (11, 12). LAA hemodynamic assessment adds incremental value (52). A TEE-derived peak LAA emptying velocity <40 cm/s predicts thromboembolic risk; <20 cm/s indicates severe stasis often associated with LAA thrombus (38). Direct LAA abnormalities detected by TEE (thrombus, spontaneous echo-contrast, low flow velocities) strongly predict thromboembolism specifically in cancer patients (18). Importantly, LAA thrombus formation has been documented even during adequate anticoagulation (e.g., lung cancer patients on warfarin or rivaroxaban), highlighting the need for dedicated LAA evaluation in this high-risk population (12). Advanced computational models improve risk stratification beyond clinical scores. Digital twin modeling integrating clinical, morphological, and hemodynamic parameters (e.g., BNP, LA volume, LAA particle count) can identify a high-risk phenogroup in which 70% of patients have a thrombosis history (19). CFD simulations combined with machine learning can generate patient-specific LAA stasis indices that also enhance risk prediction, and these tools hold promise for application in cancer-associated AF. Therefore, LAA imaging and hemodynamic evaluation are recommended before decision-making.

For patients with manageable bleeding risk and relatively preserved platelet counts, DOACs (apixaban or edoxaban) may be used. When platelet counts fall below 50 × 10⁹/L (grade 3-4 thrombocytopenia), anticoagulation management becomes challenging. In a retrospective cohort study of 131 active cancer patients with AF and platelets <50 × 10⁹/L, Drozdinsky et al. (53) reported that the 30-day incidence of arterial thromboembolism was 2% (95% CI 0.4%–7.9%) in both the anticoagulation-withheld and anticoagulation-continued groups (HR 0.92, 95% CI 0.09–9.88), while major bleeding was numerically higher in the withheld group (7.8% vs. 2.44%, HR = 3.29). These findings suggest that temporarily holding anticoagulation during periods of severe thrombocytopenia may be reasonable, given the low thrombotic event rate and the more prominent bleeding risk.

For those with markedly reduced platelet counts (<50 × 10⁹/L), prior major bleeding, or contraindications to DOACs, LAAO may be considered (12). Observational studies suggest LAAO is technically feasible in appropriately selected cancer patients with favorable outcomes, and stroke and bleeding risks are comparable to those in non-cancer populations (12). Specifically, a single-center study of 93 cancer patients (54% active cancer) reported periprocedural complication rates of 7% vs. 6% in non-cancer patients, with no significant difference in the composite endpoint of stroke, bleeding, and all-cause mortality over a median follow-up of 32 months (54). The Mayo Clinic experience (55 cancer patients, median CHA₂DS₂-VASc 5) achieved 100% procedural success, with 1-year cumulative incidences of ischemic stroke (1.4%), major bleeding (10.7%), and device-related thrombosis (4.2%); outcomes were similar to a propensity-matched non-cancer cohort (55). An Austrian multicenter registry (57 cancer patients) found no significant differences in short-term complications (5.3% vs. 7.0%) or adjusted long-term survival between cancer and non-cancer patients (56). However, a nationwide U.S. analysis of the National Inpatient Sample (2016-2019) reported higher in-hospital complication rates in cancer patients (8.8% vs. 5.7%, *p* < 0.001), primarily driven by acute kidney injury, heart failure, and cardiac tamponade, though in-hospital mortality was not increased (57). These findings indicate that while LAAO is generally safe in cancer patients, careful patient selection and periprocedural monitoring are essential, particularly in those with active malignancy or hematologic cancers.

Notably, prospective studies specifically evaluating LAA-guided anticoagulation or LAAO in cancer patients are lacking; the above recommendations are largely based on observational data and expert consensus, and decisions must be individualized (10–12, 53). A proposed algorithm for thromboembolism prevention in hemodialysis patients with AF (58) provides guidance for anticoagulation management in this specific population, but further research is needed to validate its application in other high-risk groups.

### 5.3 Post-ablation patients

The core principle of catheter ablation management is to shift from relying on LAA morphology to integrating quantitative hemodynamic and geometric parameters.

Regarding periprocedural acute risk, ablation causes acute LAA electromechanical injury, with emptying velocity dropping from 39 ± 10 to 21 ± 10 cm/s within 24 h (*p* < 0.01) and 50% of patients developing SEC (31). Chicken-wing LAA shows paradoxical risk reversal (adjusted OR = 7.2, 95% CI 2.1-24.6) (24). A meta-analysis by Papathanasiou et al. (34 studies, >8,000 patients) showed that reduced LAA emptying velocity (SMD = –0.56; 95% CI –0.73–−0.40), increased LAA volume (SMD = 0.51; 95% CI 0.35–0.67), increased orifice area (SMD = 0.35; 95% CI 0.20–0.49), and reduced LAA ejection fraction (SMD = –0.66; 95% CI –1.01–−0.32) are significantly associated with post-ablation recurrence, whereas morphological classification (chicken-wing vs. non-chicken-wing) does not predict recurrence (OR = 1.27, 95% CI 0.79–2.02, *p* = 0.32) (15). Furthermore, patients who develop incidental LAA electrical isolation during ablation face a significantly elevated risk of long-term thromboembolism, underscoring the need for continued anticoagulation or consideration of LAA occlusion (23). A pre-ablation LAA emptying velocity ≥52.3 cm/s has been identified as a predictor of decreased AF recurrence after ablation (OR = 0.55, 95% CI 0.31–0.97), indicating that higher LAA flow velocity is protective (20). Ezzeddine et al. noted that reduced emptying velocity reflects lower left atrial voltage and larger LA volume, serving as a marker of atrial remodeling (20). In long-term management, Lin et al. (27) followed 463 first-time ablation patients for one year and found that windsock LAA morphology carried the highest recurrence rate (48.3%, adjusted OR = 2.72, 95% CI 1.21-6.12). The recurrence group had significantly larger LAA orifice diameter (20.0 vs. 18.7 mm), LAA volume (14.5 vs. 13.1 mL), and LA volume (143.3 vs. 128.9 mL) (all *p* < 0.05). Notably, reduced LAA emptying velocity is a common marker for both thrombotic risk (<40 cm/s) and recurrence risk (<52.3 cm/s) (20). Digital twin studies have identified high-risk phenogroups with LAA ostial areas >500 mm², centerline lengths >50 mm, and LA volumes >150 mL (19). Therefore, for patients with windsock or cauliflower LAA and CHA₂DS₂-VASc ≥2, persistently low emptying velocity (<40 cm/s), or LA volume >150 mL, cautious continuation of anticoagulation with regular rhythm and TEE monitoring is advisable, even after successful ablation.

Consistent with the CFD/FSI findings discussed in Section 3.5, Musotto et al. demonstrated that conventional LAA morphological classification does not correlate directly with blood stasis, whereas quantitative geometric parameters (e.g., trabecular number, normalized stroke volume) are far more sensitive predictors of thrombogenic risk (28). These parameters, including the proposed blood stasis factor (BSF5), may aid in assessing thrombus stability and guiding post-ablation anticoagulation decisions, though prospective validation is lacking.

In summary, individualized management after ablation should integrate hemodynamic parameters (emptying velocity, ejection fraction, normalized stroke volume) with geometric quantitative parameters (ostial area, trabecular number, tortuosity, LA volume), combined with electrophysiological mapping, AF burden data, and digital twin models to achieve precise risk-benefit assessment.

## 6 Evidence gaps and future directions

Despite growing evidence, several important gaps remain. Current data are largely retrospective; large-scale, prospective, multicenter cohort studies are needed to validate the incremental predictive value of LAA morphological classification across different CHA₂DS₂-VASc strata under standardized anticoagulation, and to determine whether LAA morphology and function undergo dynamic changes (e.g., reverse remodeling) after ablation.

An important caveat is that the associations between LAA morphology and thromboembolic outcomes discussed in this review are primarily observational and should not be interpreted as causal. While computational models demonstrate biological plausibility by simulating thrombus formation in specific anatomical regions, these in silico findings require experimental and clinical validation. Prospective studies with standardized imaging protocols and longitudinal follow-up are needed to establish whether morphological features directly drive thrombogenesis or serve as markers of underlying atrial pathology (e.g., fibrosis, remodeling).

An additional unresolved question is whether LAA morphology remains stable over time or undergoes remodeling during the natural history of AF, particularly in patients with long-standing persistent or permanent AF. Chronic AF is associated with progressive atrial dilatation and fibrosis, and the LAA may undergo analogous structural changes. If morphological changes occur, for example, due to progressive atrial dilation, fibrosis, or changes in wall compliance, this could have important implications for the clinical applicability of morphology-based risk stratification, as a single baseline assessment might not reflect future risk. Conversely, if morphology is determined by embryological development and remains stable, a one-time assessment may suffice. Serial imaging studies (CT or MRI) with quantitative morphological analysis are needed to address this question. Additionally, the potential for reverse remodeling after successful ablation or rhythm control, and its effect on LAA morphology and function, remains unexplored.

The digital twin approach requires external validation in larger, more diverse populations with standardized CFD modeling before an integrated risk score (incorporating LAA subtypes, quantitative geometry, hemodynamics, AF burden, and clinical factors) can be prospectively tested against traditional scores. No randomized controlled trial has compared imaging-guided versus clinical-score-guided anticoagulation; dedicated RCTs with composite endpoints (stroke, systemic embolism, major bleeding, all-cause mortality) are urgently needed. Causal machine learning and digital twins are complementary; hybrid models using machine learning to pre-screen patients for digital twin simulation could reduce computational burden, but prospective validation is lacking. Additional research directions include the interaction between AF burden and LAA parameters, the dynamic recovery of LAA function post-ablation, mechanisms of paradoxical embolization after anticoagulation initiation in long-standing persistent AF, and the potential relationship between LAA morphology (e.g., windsock type) and Warfarin ridge anatomy affecting ablation outcomes. Finally, standardization of imaging protocols (CT, MRI, TEE) and quantitative parameter definitions, along with AI-driven automated analysis, are essential to reduce interobserver variability and enable multicenter collaboration. The limitations of this narrative review, including its non-systematic nature, potential selection bias, heterogeneity of included studies, and the preliminary status of digital twin research, should be acknowledged when interpreting its findings.

## 7 Conclusion

Traditional CHA₂DS₂-VASc and CHA₂DS₂-VA scores have well-recognized limitations, particularly in low-score patients who nevertheless thrombose, high-score event-free individuals, cancer patients, and post-ablation cohorts. A common reason is the neglect of the LAA, the source of >90% of thrombi in non-valvular AF. LAA morphological subtypes correlate with long-term stroke risk, but the relationship is context-dependent: chicken-wing is protective in the long term yet paradoxically increases peri-ablation risk, and windsock morphology predicts post-ablation recurrence. Importantly, conventional morphological classification does not correlate directly with blood stasis (*p* = 0.365); quantitative geometric parameters (trabecular number, normalized stroke volume, ostial area) and hemodynamic indices are far more sensitive predictors. Digital twin technology, integrating multimodal clinical, morphological, and hemodynamic data, can identify a high-risk phenogroup with a 70% thrombus history rate. Based on these findings, we propose a practical two-tier assessment framework: basic morphological classification for initial screening, followed by multimodal refined evaluation (digital twin) for intermediate-/high-risk or clinically challenging cases. This framework represents a paradigm shift from static clinical scores toward mechanism-driven, individualized risk management, but should be viewed as hypothesis-generating and requires prospective validation.
